# Measuring human rights violations from an ecological perspective using a locally generated instrument: a cross-sectional study of Palestinians in the Israeli-occupied West Bank

**DOI:** 10.3389/fpubh.2025.1557817

**Published:** 2025-05-22

**Authors:** Rita Giacaman, Rula Ghandour, Weeam Hammoudeh

**Affiliations:** Institute of Community and Public Health, Birzeit University, Birzeit, Palestine

**Keywords:** human rights violations, community and society, government, Israeli military occupier, measurement, West Bank, Israeli occupied Palestinian territory

## Abstract

**Introduction:**

This study presents findings from a cross-sectional household survey conducted among Palestinians in the Israeli-occupied West Bank to assess the reported prevalence of human rights violations committed by various potential perpetrators.

**Methods:**

We used a context-specific tool developed from the ground up using qualitative methods to enhance our understanding of what Palestinians consider to be human rights violations. This tool aligns with our conceptualization of potential perpetrators, which includes the family, the community, the Palestinian Authority, and the Israeli military occupier and colonizer of Palestinian land.

**Results:**

Overall, as many as 60% of participants reported being exposed to one or more human rights violations, with the most frequently reported being restrictions on mobility, safety, freedom, and the exercise of political rights. Regression analysis revealed that women were more likely to report violations perpetrated by the family compared to men, whereas men were more likely to report violations by the Palestinian Authority and the Israeli military occupier. Palestinians living in Area C, fully controlled by Israel and where illegal Israeli settlements on confiscated Palestinian land are located, had higher odds of reporting experiences of general human rights violations, alongside those committed by the Israeli military occupier, the Palestinian Authority, and the family. Participants with lower educational levels and those from poorer backgrounds had higher odds of reporting human rights violations by all offenders.

**Discussion:**

This study underscores the importance of considering the family and community as potential human rights perpetrators and highlights the significance of using mixed methods in research to ground findings in participant experiences. Particularly during wartime, as violence permeates daily life, the combination of violations from family, community, government, and military occupiers is likely to be synergistic, exacerbating the experienced suffering and making life increasingly difficult to endure. This may also lead to significant negative impacts on health, whether physical or mental, as health is fundamentally a social and political construct.

## Introduction

The concept of human rights is complex and difficult to assess (1). It is a multidimensional concept (2) and contains different facets. It is based on the principles of freedom, including political, economic, and social freedoms, equality, and fairness (3). Nevertheless, it is insufficiently conceptualized, with subjective dimensions and subtle variations in the different understandings of the concept, making measurement difficult (4). Notably, global indicators fail to consider the socioeconomic and political conditions of various countries, where human rights and their violations occur across a range of contexts and cultures, rather than being a uniform concept applicable universally (5).

A substantial body of literature exists on human rights violations outlined by the Universal Declaration of Human Rights (UDHR) and International Humanitarian Law (IHL), applicable to various countries and peoples. This includes, for example, the Rohingya of Myanmar, who have endured years of systematic human rights violations (6), as well as Afghanistan, where decades of prolonged conflict have involved mass human rights violations perpetrated by various parties, including the United States (7). However, such literature typically emphasizes indicators, measurements, and data collection related to violations committed by armies occupying territories in the midst of international conflicts, as well as the rights of citizens impacted by military occupiers and governments and their human rights practices (8, 9). In this regard, the focus tends to be on military occupiers and/or national governments rather than also considering other potential perpetrators of human rights violations, including families and communities where people live.

At the same time, available human rights data generally relies on annual reports from human rights organizations and governments (10, 11). Such data are usually retrieved from public documentation, which has incomplete coverage of the list of internationally recognized rights (12), or from events found in formal legal documents, records of complaints, socioeconomic and administrative statistics from government and civil society, declared commitments to protect rights as written in national constitutions, and the degree to which countries are parties to human rights treaties over time (13, 14). In fact, most cross-national data sets focusing on civil and political rights are sourced from English-language secondary sources. This approach under-represents the level of violations globally and is described as biased under-reporting, making it difficult to compare across countries and cultural contexts (15). These observations raise questions about the accuracy and completeness of the collected information, the relevance of such information to specific settings, and, significantly, the omission of human rights violations occurring at the family and community/societal levels.

The existing English language literature indicates that efforts have been made to create more accurate and comprehensive measures of human rights violations and that select measures have been developed. However, once again, such measures primarily focus on the compliance of governments with international treaties and obligations, along with what are termed performance indicators, detailing the status of civil and political rights within a population (16, 17). This includes two measures known as the political terror scales, which utilize data from Amnesty International and the U.S. Department of State to examine governments’ economic, political, and cultural conditions of human rights observance for cross-national comparisons (11). Another scale developed to rank human rights achievements on a scale of 0–100 is the Humana Index, which highlights governmental policies and practices related to citizens’ civil and political rights—specifically, civil and political liberties—(18) and their link to governmental performance.

Additional initiatives aimed at developing measures for human rights violations include the Human Rights Measurement Initiative (HRMI), which also focuses on national government practices (12). This initiative highlights the lack of attention to economic and social rights and identifies five rights specified in the International Covenant on Economic, Social, and Cultural Rights: the rights to food, education, health, housing, and decent work. However, it is recognized that other rights contained in various human rights treaties have not been addressed. The authors also argue that human rights data are underutilized due to the reliance on public documentation as the information source and the barriers to data access faced by individuals outside of academia, among other reasons. They employ what is known as ‘human-centered design’ by involving key user groups, including human rights researchers and practitioners, and seeking their feedback and engagement in developing indicators. This initiative appears to currently focus on measuring civil and political human rights (19). However, because objective and comprehensive data do not exist, the Initiative gathers information from human rights researchers and practitioners who monitor events in their countries through an expert opinion survey. Overall, although efforts have been made to quantify trends in human rights violations, existing human rights indicators are described as insufficient and problematic, often overlooking local realities. This highlights the need for an alternative strategy in designing bottom-up and contextually embedded human rights indicators (20).

### The Israeli military occupation and the human rights violations of the Palestinian Authority in the occupied Palestinian territory

The West Bank and the Gaza Strip are part of the Palestinian territory occupied by Israel in 1967 during the 6-day Israeli war (21). In 1993, the Oslo Accords between the Palestinian Liberation Organization and Israel were signed with the intention of ending the Israeli occupation of Palestinian land (the West Bank, including Palestinian East Jerusalem, and the Gaza Strip) and the creation of an independent Palestinian state. However, instead of achieving these aspirations, the Accords resulted in the further entrenchment of Israeli occupation and the institutionalization of apartheid. The West Bank was consequently divided into three main areas: Area A, covering approximately 10% of West Bank land, with supposed control by the Palestinian Authority; Area B, covering approximately 30% of West Bank land, with Palestinian Authorities’ civil control and joint Israeli-Palestinian security control; and Area C, which included approximately 60% of West Bank land with full Israeli control, including security, planning, and construction (22). It is worth noting that areas A, B, and C do not have clear borders and are widely fragmented, overlapping, and spread over the West Bank, with Area A separated from other A areas, as well as B by C areas, disrupting the continuity of Palestinian Authority control over land and allowing the Israeli military to control the movement of people and goods as well as resources (23). Since then, the Israeli military occupier has continued to control movement, borders, water, and other resources, in addition to the total control over Area C and the main roads connecting different West Bank governorates (22), and the building of illegal Israeli settlements on confiscated Palestinian land (24, 25).

Human rights violations contravening the Geneva Conventions committed by Israel against Palestinians living under Israeli military rule and colonization are monitored and reported by United Nations (UN) agencies, Human Rights Watch, Amnesty International, and several other bodies. Yet despite such reports, Israel continues to act with impunity, and Palestinians continue to suffer daily. Human rights violations include exposure to political and structural violence endured by the population of both the West Bank and the Gaza Strip (and especially during the Gaza Strip genocide beginning October 2023) (65), with Palestinian land confiscation by the Israeli occupier, the building of illegal Israeli settlements on Palestinian land, and colonization of the occupied Palestinian territory recently described as Apartheid by Palestinians (26) and other international groups such as Amnesty International (27) and other human rights organizations. Serious human rights violations are prescribed by a variety of Israeli military orders to restrict the movement of people and goods while enforcing sudden closure policies and a heavy bureaucracy controlling daily life, other than direct exposure to political violence among persons and groups, including imprisonment, injury, disability, and death (28, 29). Such violations are perpetrated by both the Israeli army and Jewish settlers (28), who continue to illegally build settlements on confiscated Palestinian land despite the Oslo Accords of 1993 prohibiting the establishment of new settlements (30).

Since 7 October 2023, according to Amnesty International: “Israel has unleashed hell and destruction on Palestinians in Gaza brazenly, continuously and with total impunity.” Furthermore, Amnesty concluded that Israel has committed acts that are prohibited under the Genocide Convention and that what is being committed by Israel against Gazans is genocide (31, 65). Ironically, and although these pale in the face of Israeli violations of rights, what adds insult to injury are the human rights violations of the Palestinian Authority itself against its Palestinian citizens, which are also beginning to be reported in the literature (32). Violations include rampant corruption, negatively affecting all aspects of daily life, including the violation of basic human rights where corruption and cronyism are reportedly used to, for example, limit access to employment opportunities and scholarships for students (33) or the restriction of public discourse impeding free speech (34); imprisonment and mistreatment of detainees, and political repression, among other violations (35, 36). Indeed, the Palestinian Authority has consistently failed to protect Palestinians from Israeli military occupation and has, in effect, become a protector of the Israeli occupier given massive donor investments, and with security collaboration between Israel and the Palestinian Authority allowing Israel to fulfill its colonial ambitions while pursuing so-called peace (32).

Nevertheless, these are not the only violations of human rights that Palestinians under Israeli occupation endure; human rights are also violated by families as well as communities, where various factors can contribute to violence and other human rights abuses (36, 37). Unfortunately, violations by families and communities have not received due attention in research and interventions, except perhaps for the misconceptualized issue of domestic or what is called (intimate partner) as we have a real problem with the concept intimate partner violence, which almost always focuses only on women (38, 39), as if Palestinian men are, by definition, perpetrators of violations. Furthermore, domestic violence is treated in isolation from the broader violence surrounding the family and how such broader-level violence affects the presence and extent of domestic violence (40).

Overall, this research defines human rights violations through an ecological framework that positions individuals, both men and women, within family units and subsequently within their communities or society. This framework allows for the potential that either individuals or the community may be responsible for violating the human rights of others within the population, alongside the violations arising from Israeli occupation and the actions of the Palestinian Authority. Simultaneously, human rights indicators must be measurable; they should relate to the local context and be supported by qualitative data to help contextualize the indicators for various situations. These observations highlight the need for developing contextually and socio-culturally relevant tools to accurately measure human rights violations. This includes specific contexts such as the West Bank, an area of Israeli-occupied territory that has endured over 57 years of Israeli military rule, colonization, and chronic, pervasive human rights violations committed against persons, groups, and the entire Palestinian population.

In this study, we used the results of a cross-sectional household survey conducted in the Israeli-occupied West Bank to assess the reported prevalence of human rights violations among Palestinian adults, which have been reported or to have been committed by various entities, employing a context-specific tool that was developed for this purpose. We hope that this study can provide significant insights into measuring the human rights violations experienced by Palestinians while contributing to the broader discourse and research on human rights violations globally, particularly in war-like conditions and conflict-affected zones.

## Materials and methods

This study presents the findings of a human rights violations study based on a cross-sectional household survey conducted among Palestinians living in the West Bank of the Israeli-occupied Palestinian territory in 2022. The target population included all Palestinians aged 18 and above living in the West Bank at the time of the survey. The sampling frame included all population locales in the West Bank, as published by the Palestinian Central Bureau of Statistics (PCBS). A probability sample of 2000 households was selected using a stratified multistage cluster sampling technique. The sample was first stratified by governorate (11 in total), and then clusters were chosen within each governorate using the probability proportional to size (PPS) technique. A total of 108 clusters were randomly selected. Within each cluster, households were chosen using a random walk method, beginning from a randomly identified landmark and then including every fifth household. Finally, one adult per household was randomly selected using a Kish table, ensuring a representative sample of the target population. If the selected individual was not at home, an appointment was made up to three times. Then, he or she would be excluded from the sample. Only one questionnaire per household was collected. Data were collected by trained field workers. The final sample comprised 2003 individuals, achieving a response rate of 95%.

The study instrument included a locally developed scale that measures human rights violations committed by various perpetrators in accordance with our conceptual framework, which considers the family, the community/society, the Palestinian Authority, and the Israeli occupier of Palestinian land as potential perpetrators. The human rights scale was created based on qualitative assessments, which helped identify what Palestinians in the West Bank recognize as human rights violations (33). This scale was subsequently piloted quantitatively, with factor analysis conducted to identify and determine the key dimensions of these reported human rights violations. The resulting scale consists of 15 items measured using the Likert scale. For each item, respondents were asked to specify who the perpetrator was. Four main perpetrators were identified: the family, the community, the Palestinian Authority, and the Israeli military occupier of Palestinian land. Additionally (33), other demographic, socioeconomic, and political variables were also collected.

The main outcomes of this study are the human rights violations identified by the locally developed scale, which inquires about human rights violations across various dimensions from individuals living in diverse contexts. We constructed five different Human Rights Violation (HRV) scales. The first scale is the “General HRV scale,” which assesses human rights violations from any perpetrator, while the remaining four scales, the “Specific HRV scales,” evaluate violations by specific perpetrators within the socio-ecological framework described above. Cronbach’s alpha was calculated to evaluate the reliability of the scales. All scales demonstrated good internal consistency, as evidenced by their Cronbach’s alpha values. The “General HRV scale” had an alpha of 0.87. The specific HRV scales also showed strong internal consistency, with Cronbach’s alpha values of 0.93 for violations by the family, 0.82 for violations by the community, 0.91 for violations by the Palestinian Authority, and 0.89 for violations by the Israeli military occupation. We then recoded the scores of the five different scales as categorical variables, with 0 indicating no violation and 1 indicating the presence of at least one violation (please refer to Supplementary material 1 for scale items and scoring procedure).

Other key variables included participant demographic and socioeconomic characteristics. Among the demographic factors, we collected data on age, gender, and governorate of residence, which we recorded in the North, Center, and South regions of the West Bank, according to the classifications provided by the Palestinian Central Bureau of Statistics. We also included the locality where people lived, categorized as urban, rural, or Palestinian refugee camp, with refugee camps having been established to accommodate Palestinian refugees from the 1948 and 1967 Arab-Israeli wars, who were dispossessed and displaced due to the establishment of the State of Israel in 1948 and the occupation of the West Bank, including East Jerusalem, and the Gaza Strip by Israel in 1967. Additionally, we asked respondents to classify their place of residence based on the area designations outlined in the Oslo Accords between the Palestine Liberation Organization and Israel: Areas A, B, or C (23). Marital status, employment status, and participants’ reported economic status in relation to others around them were also gathered as proxies for socioeconomic status. Finally, to assess the level of disability or functional difficulty among the study participants, we utilized the short set of the Washington Group questionnaire on disability survey (41).

### Statistical analysis

We used univariate analysis to describe the characteristics of study participants and the reported human rights violations in general, as well as by different perpetrators. Multiple logistic regression models were conducted for the five human rights violation scales. In all models, the dependent variable was the binary variable for human rights violations, and the independent variables included age, sex, West Bank region, locality, area classification according to the Oslo agreement (i.e., area A, B, or C), marital status, education, employment, reported economic status, and disability status. All variables were entered into the model simultaneously, regardless of their significance level in the bivariate analysis. The first regression model, referred to as the general model (model 1: GHRV), included the general human rights violation scale as the primary outcome. The other four models substituted the general human rights violation scale with one of the four specific scales, namely family, community, Palestinian Authority, and Israeli military occupier. The goodness-of-fit tests for all models confirmed that the logistic regression models adequately fit the data, with classification accuracy exceeding 62% across all models. Data analysis was conducted using Stata version 18.

### Ethical considerations

We obtained ethical approval for this study from the Institute of Community and Public Health at Birzeit University’s Research Ethics Committee (Ref. No. 2022 (9–1)), ensuring compliance with all relevant ethical principles and guidelines. Oral consent was obtained from study participants after providing them with comprehensive information about the study’s objectives, procedures, potential risks, and benefits. Obtaining oral consent from participants is an appropriate and approved method for this population and aligns with the Birzeit University Research Ethics Committee Guidelines, as people locally tend to feel uneasy about signing consent forms and prefer oral consent instead. Participants were assured of their right to withdraw from the study at any time without adverse consequences. Confidentiality and anonymity were strictly maintained, with all personal information and data treated confidentially and accessible only to the research team for research purposes.

## Results

We interviewed 2,003 people, of whom 89 did not answer the human rights violation questions regarding the prevalence of violations, leaving a total of 1,914 participants for the final analysis. The gender distribution was nearly balanced, with 49.2% men and 50.8% women. The mean age of the participants was 40.6 ± 15.4 years, ranging from 18 to 93 years. Regarding place of residence, 44.5, 20.0, and 35.4% were living in the northern, central, and southern regions of the West Bank, respectively. Concerning locality type, 59.6% resided in urban areas, 33.3% in rural settings, and 7.0% in Palestinian refugee camps. The majority (68.4%) lived in Area A, while 12.2% resided in Area B and 19.4% in Area C.

Of the total, 69.3% were married, 24.5% were single, 2.0% were divorced or separated, and 4.3% were widowed. For educational attainment, 48.6% had an education level below high school (the governmental high school *Tawjihi* certificate), 21.0% had completed and passed the *Tawjihi* examination, and 30.4% had completed at least some post-secondary education. More than half of the participants (53.6%) were not working at the time of the survey, either unemployed or for other reasons, such as being housewives, students, retired, or disabled. Furthermore, 53.9% of study participants reported having a good economic status compared to others around them; 27.6% of participants rated their economic status as very good to excellent, while 18.5% rated it as fair to poor. Lastly, the results indicated that 11.8% of participants reported having at least one functional difficulty according to the Washington Group classification (Table 1).

**Table 1:** Sample characteristics (*N* = 1914).

		*N*	%
Age	18–24	338	17.7%
	25–34	457	23.9%
	35–44	401	21.0%
	45–54	312	16.3%
	55–93	409	21.1%
Sex	Male	942	49.2%
	Female	972	50.8%
West Bank Region	North	851	44.5%
	Center	385	20.1%
	South	678	35.4%
Locality type	Urban	1,142	59.6%
	Rural	638	33.3%
	Refugee camp	134	7.0%
Classification of the area according to the Oslo Accords	A	1,302	68.4%
	B	233	12.2%
	C	369	19.4%
Marital status	Married	1,326	69.3%
	Single	468	24.5%
	Separated/divorced	38	2.0%
	Widowed	82	4.3%
Educational attainment	Less than high school (*tawjihi*)	930	48.6%
	Passed high school (*tawjihi*)	401	21.0%
	Completed college or higher education	583	30.4%
Work	No^*^	1,026	53.6%
	Yes	888	46.4%
Reported economic status compared to other	Very good to excellent	527	27.6%
	Good	1,031	53.9%
	Fair to poor	354	18.5%
Disability status	None	1,688	88.2%
	At least one	226	11.8%

* Student, housewife, retired, disabled, unemployed, and so on.

**Figure 1:**
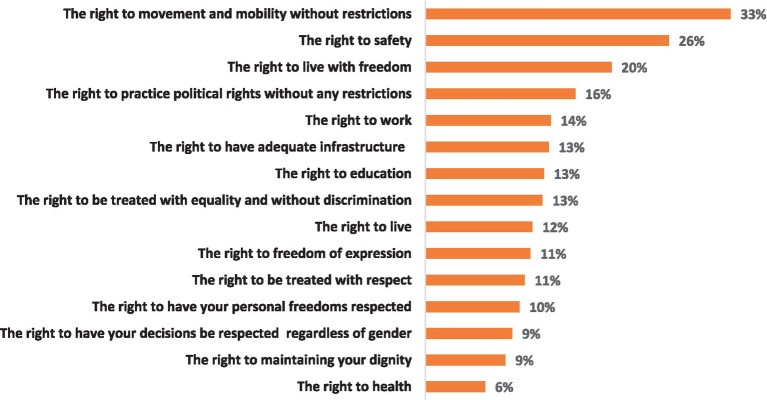
Prevalence of different Human Rights Violations scale items in the general human rights violation scale (GHRV) * (*N* = 1914). *These are the general human rights violation levels regardless of their perpetrators.

Figure 1 illustrates the distribution of the 15 items on the GHRV scale, regardless of the perpetrator. The highest level of violation was reported for the right to movement and mobility without restrictions, with 33% of respondents identifying it as a human rights violation they experienced by being unable to move freely. The right to safety was the second most violated right, reported by 26% of participants. The right to live with freedom was cited as violated by 20% of respondents. Among the total sample, 16% of respondents indicated that the right to practice political rights without any restrictions was being violated. The right to work was reported as violated by 14% of respondents, as they were unable to find employment. Three rights were equally reported as violated by 13% of people: the right to adequate infrastructure, the right to education, and the right to be treated equally without discrimination. The right to live was reported as violated by 12% of respondents. Both the right to freedom of expression and the right to be treated with respect were each reported as violated by 11% of individuals. The right to have personal freedoms respected was viewed as violated by 10%. Furthermore, the right to have personal decisions respected regardless of gender and the right to maintain personal dignity were each noted as violated by 9% of individuals. Finally, the right to health was reported as the least violated, with 6% of respondents indicating that this right was violated.

**Figure 2:**
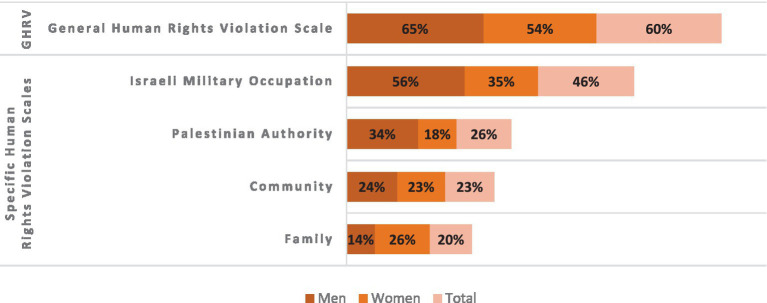
Distribution of human rights violations prevalence across general and four specific scales by gender (*N* = 1914).

Figure 2 presents the prevalence of HRV in general and among different perpetrators categorized by gender. This figure indicates that 60% of the study participants reported experiencing at least one human rights violation by any perpetrator, with higher percentages among men (65%) compared to women (54%). In terms of specific HRV scales, the prevalence was 46, 26, 23, and 20% for violations of human rights by the Israeli military occupation, the Palestinian Authority, the community, and the family, respectively. Women reported higher levels of HRV from the family, while men reported greater HRV from the Israeli military occupation and the Palestinian Authority, with both men and women reporting similar levels of HRV from the community.

The multivariate logistic regression analysis revealed that several demographic and socioeconomic variables were significantly associated with reports of experiencing Human Rights Violations (HRV) by various perpetrators. Age was significantly associated with HRV on the General HRV scale, as well as in both HRV reports by family and by the Israeli military occupation. For each 1-year increase in age, there was approximately a 20% increase in the odds of being exposed to GHRV (OR = 1.2, 95% CI: 1.1–1.3, *p* < 0.001). Age was also positively associated with HRV reported by family (OR = 1.1, 95% CI: 1.0–1.2, *p* < 0.05) and by the Israeli military occupation (OR = 1.1, 95% CI: 1.1–1.2, *p* < 0.01), but not with community or Palestinian Authority reports.

Sex differences in HRV were pronounced across multiple perpetrators. Men were 1.4 times more likely to report GHRV compared to women (OR = 1.4, 95% CI: 1.1–1.8, *p* < 0.01). Notably, men were significantly less likely to experience HRV from family members compared to women (OR = 0.4, 95% CI: 0.3–0.6, *p* < 0.001), while they were more likely to experience HRV from other perpetrators. Men were 2.2 times more likely to report HRV from the Palestinian Authority (OR = 2.2, 95% CI: 1.6–2.9, *p* < 0.001) and twice as likely to report it from Israeli military occupation (OR = 2.0, 95% CI: 1.5–2.5, *p* < 0.001) compared to women, while no significant variation by sex was observed in HRV from the community.

Individuals living in the northern West Bank were 1.4 times more likely to report HRV by the community (OR = 1.4, 95% CI: 1.1–1.9, *p* < 0.05) and 1.6 times more likely to report HRV by the Palestinian Authority (OR = 1.6, 95% CI: 1.1–2.1, *p* < 0.01) compared to those residing in the central West Bank. Furthermore, individuals in the southern West Bank were 1.5 times more likely to report HRV by the Palestinian Authority (OR = 1.5, 95% CI: 1.1–2.1, *p* < 0.05) compared to other regions of the West Bank. No other variations in HRV experiences by region were identified at the family and Israeli military occupation levels.

Living in Area C was significantly associated with higher odds of experiencing HRV from different perpetrators. Except for HRV related to the community, residents of Area C were more likely to report experiencing HRV compared to those in Area A, with (OR = 1.5, 95% CI: 1.2–1.9, *p* < 0.01) for the general HRV scale, (OR = 1.4, 95% CI: 1.0–1.9, *p* < 0.05) for HRV by family, (OR = 1.7, 95% CI: 1.3–2.3, *p* < 0.001) for HRV by the Palestinian Authority, and (OR = 1.6, 95% CI: 1.2–2.0, *p* < 0.001) for HRV due to Israeli military occupation.

Only those who were separated from their spouses or divorced were significantly more likely to report HRV from the family (OR = 2.7, 95% CI: 1.4–5.5, *p* < 0.01) compared to those who reported being married. For HRV reported by the family, lower education levels were associated with higher odds of exposure to HRV. Participants with less than a high school education (*tawjihi*) were 2.3 times more likely to report HRV from the family (OR = 2.3, 95% CI: 1.6–3.1, *p* < 0.001) compared to those who reported having post-secondary education. Similarly, those who passed their *tawjihi* but did not pursue post-secondary education were 1.7 times more likely to report HRV from the family (OR = 1.7, 95% CI: 1.2–2.5, *p* < 0.01) compared to those who reported having a college or post-secondary education.

In contrast, positive relationships were found between education and HRV by the Palestinian Authority and the Israeli military occupation. Those who reported having less than a high school education were 0.6 times as likely to report HRV by the Palestinian Authority and 0.7 times as likely to report HRV by the Israeli military occupation compared to those who reported having post-secondary education (OR = 0.6, 95% CI: 0.5–0.8, *p* < 0.001 and OR = 0.7, 95% CI: 0.6–0.9, *p* < 0.01, respectively).

Work was significantly associated with HRV solely due to the Israeli military occupation. Those who reported being employed at the time of the survey were 1.3 times more likely to report HRV linked to Israeli military occupation compared to those who were unemployed (OR = 1.3, 95% CI: 1.0–1.7, *p* < 0.05). Interestingly, reported economic status also showed a significant association with HRV. Individuals who described their economic status as good compared to those around them were 1.5 times more likely to report HRV on the general scale, 1.4 times more likely to report HRV from the community, and 1.4 times more likely to report HRV due to the Israeli military occupation, compared to those who rated their economic status as very good to excellent, with no statistical significance for HRV related to family or the PA. The link between economic status and HRV becomes more pronounced for those identifying as fair to poor. Those reporting fair to poor economic status were 2.6 times more likely to report HRV on the general scale, 2.2 times more likely for HRV related to family, 2.7 times more likely for HRV from the community, 1.7 times for the Palestinian Authority, and 1.8 times for HRV due to Israeli military occupation when compared to individuals with very good to excellent economic status relative to those around them.

Finally, the respondents’ disability status was associated with increased reports of HRV, primarily from family members. Participants with at least one disability were 1.4 times more likely to report HRV on the general scale and 1.5 times more likely to report HRV through family sources compared to those who reported not having any disabilities (OR = 1.4, 95% CI: 1.0–2.0, *p* < 0.05). However, this relationship was not significant in the other HRV scales (see Table 2).

**Table 2:** Multiple logistic regression analysis for the GHRV scale and the four specific human rights violation scales.

		General HRV	HRV by the family	HRV by the community	HRV by the PA	HRV by the occupation
		AOR (95%CI)	AOR (95%CI)	AOR (95%CI)	AOR (95%CI)	AOR (95%CI)
Age (continuous)		1.2 (1.1–1.3) ***	1.1 (1.0–1.2)*	1.0 (0.9–1.1)	1.0 (0.9–1.1)	1.1 (1.0–1.2)**
Sex	Women	Ref	ref	ref	ref	ref
	Male	1.4 (1.1–1.8)**	0.4 (0.3–0.6)***	1.0 (0.7–1.3)	2.2 (1.6–2.9)***	2.0 (1.5–2.5)***
Region	Center WEST BANK	ref	ref	ref	ref	ref
	North	0.8 (0.6–1.1)	1.4 (1.0–2.0)	1.4 (1–1.9)*	1.6 (1.1–2.1)**	0.8 (0.6–1.1)
	South	0.9 (0.7–1.2)	1.3 (0.9–1.8)	1.4 (1–1.9)	1.5 (1.1–2.1)*	0.8 (0.6–1.0)
Locality	Urban	ref	ref	ref	ref	ref
	Rural	1.2 (1.0–1.5)	1.1 (0.8–1.4)	1.1 (0.9–1.4)	1.2 (0.9–1.5)	1.2 (1.0–1.5)
	Camp	1.0 (0.7–1.5)	0.9 (0.5–1.4)	0.7 (0.4–1.1)	0.9 (0.6–1.4)	1.0 (0.7–1.4)
Area	Area A	ref	ref	ref	ref	ref
	Area B	1.2 (0.9–1.6)	0.9 (0.6–1.4)	1.2 (0.9–1.7)	1.4 (1.0–2.0)	1.2 (0.9–1.6)
	Area C	1.5 (1.2–1.9)**	1.4 (1.0–1.9)*	1.1 (0.8–1.5)	1.7 (1.3–2.3)***	1.6 (1.2–2.0)***
Marital status	Married	ref	ref	ref	ref	ref
	Single	1.1 (0.8–1.4)	1.1 (0.8–1.5)	1.3 (0.9–1.7)	1.0 (0.7–1.3)	1.0 (0.8–1.3)
	Separated/divorced	1.3 (0.6–2.7)	2.7 (1.3–5.5)**	0.8 (0.3–1.7)	1.0 (0.4–2.3)	1.2 (0.6–2.4)
	Widowed	0.9 (0.5–1.5)	1.2 (0.7–2.1)	0.8 (0.4–1.4)	1.0 (0.5–1.8)	1.1 (0.6–1.7)
Education	Post-secondary education	ref	ref	ref	ref	ref
	Less than high school (*tawjihi*)	0.9 (0.7–1.1)	2.3 (1.6–3.1)***	1.0 (0.8–1.3)	0.6 (0.5–0.8)***	0.7 (0.6–0.9)**
	Passed high school (*tawjihi*)	1.1 (0.8–1.5)	1.7 (1.2–2.5)**	0.8 (0.6–1.1)	0.7 (0.5–0.9)*	1.1 (0.8–1.4)
Work	No	ref	ref	ref	ref	ref
	Yes	1.2 (0.9–1.5)	0.9 (0.6–1.2)	1.0 (0.8–1.3)	1.1 (0.8–1.4)	1.3 (1.0–1.7)*
Reported economic status	Very good to excellent	ref	ref	ref	ref	ref
	Good	1.5 (1.2–1.8)***	1.3 (0.9–1.7)	1.4 (1.0–1.8)*	1.3 (1.0–1.7)	1.4 (1.1–1.7)**
	Fair to poor	2.6 (1.9–3.6)***	2.2 (1.5–3.2)***	2.7 (2.0–3.8)***	1.7 (1.2–2.4)***	1.8 (1.3–2.4)***
Disability	None	ref	ref	ref	ref	ref
	At least one	1.4 (1.0–2.0)*	1.5 (1.1–2.2)*	1.3 (0.9–1.9)	1.3 (1.0–1.9)	0.9 (0.7–1.3)

*p < 0.05, **p < 0.01, ***p < 0.001. AOR: adjusted odds ratio (95% confidence interval).

## Discussion

This study presents findings from a cross-sectional analysis of the adult Palestinian population living in the Israeli-occupied West Bank. We assessed the prevalence of human rights violations among adult Palestinians from an ecological perspective using a locally developed research tool. The results highlight human rights violations committed by four main perpetrators: the family, the community, the Palestinian Authority, and the Israeli military occupier and colonizer of Palestinian land.

Overall, approximately 60% of participants in this study reported that their human rights had been violated by any of the four aforementioned perpetrators. This indicates a very high level of exposure to human rights violations, with over half of the participants being women (54%) and more than two-thirds of the men (65%) stating that they have experienced a violation of their rights at least once. Unfortunately, such results would be challenging to compare with other reports, given our specific conceptualization of human rights violations, which includes perpetrators beyond just the government or military occupier. Additionally, our sample represents the entire population, contrasting with other published reports where data were derived from records that seem to only uncover the tip of the iceberg. Nonetheless, we anticipate conducting studies that utilize the ecological conceptualization and the same instrument to enable comparison of results across similar and diverse populations.

Significant findings from this research indicate that the highest reported level of human rights violations by any perpetrator relates to the right to mobility without restriction, followed by safety, freedom, and the exercise of political rights without restriction at 33–16%, respectively. In the Palestinian context, these rights are largely violated due to the Israeli occupier, but ironically also by violations committed by the Palestinian Authority. Such violations have been repeatedly documented, albeit without accurately quantifying the proportion of the affected population or providing generalizable demographic data (28, 42–44). To be clear, the human rights violations related to the political context are part of the lived experience of Palestinians under Israeli occupation and colonization, as evidenced by the results of this study.

The violation of the rights to work, to live in an area with adequate infrastructure, to education, and to health was also reported by 6–14% of respondents, likely indicating that the economic and social rights of Palestinians in the West Bank are being breached, with such results consistent with other reports as well (12). The right to life, freedom of expression, personal freedom, respect for personal decisions regardless of gender, and the maintenance of one’s dignity were also reported by 9–12% of respondents, all of which could be understood as part of civil and political rights. Indeed, such violations have been documented in the literature, although the findings (45, 46) may not necessarily be representative of the entire Palestinian population living in the West Bank.

Regression analysis consistently indicated that older persons tend to experience more violations due to a lifetime of accumulated experiences. Furthermore, men were more likely to report exposure to GHRV than women, whereas women reported greater odds of facing violations from their families than men did. In contrast, men indicated higher odds of violations perpetrated by Palestinian Authority and the Israeli military. Though both genders experience violence and oppression under settler colonialism, Palestinian women face a triple oppression: violence from the Israeli army and settlers, the impact of patriarchy and its associated policies, and socio-legal discrimination, which encompasses the uneven treatment of women influenced by social and legal factors (47). However, women’s freedoms and their ability to move outside the home are restricted by patriarchal relations, with women typically being confined at home compared to men (48). In contrast, men enjoy the freedom to go out but bear the societal burden of being the breadwinners for their families, necessitating their search for work as part of their defined role. Consequently, they are more exposed to broader political violations than women (49). It is important to consider the relationship between exposure to political violence and rights violations at home in research on domestic violence, as one type of violation can influence and trigger another.

Variation by district residency was also significant. Northern West Bank Palestinians were more likely to report violations by both the community and the Palestinian Authority compared to those living in the central West Bank. Similarly, persons living in the southern West Bank were also more likely to report Palestinian Authority related violations. While the community violations in the north remain unexplained and require further research, violations associated with the Palestinian Authority are understandable, as support and services are generally more accessible in the central region where the Palestinian Authority government and institutions are predominantly located compared to other areas (50). At the same time, residents of Area C, which is completely controlled by the Israeli military and contains illegal Israeli settlements on Palestinian land, were found to have higher odds of reporting experiences of general human rights violations by the Palestinian Authority, the Israeli military occupier, and their families. Such results are expected, as Area C in the West Bank exhibits the strongest effects of colonization, characterized by islands that are isolated from each other and severely restrict mobility. The Palestinian population faces chronic exposure to land confiscation, control of water sources, and other forms of direct and indirect violence (51), while the Palestinian Authority has no control over the area (52) and is unable to meet the basic needs of the local population.

The results indicate that the lower the education level of participants, the higher the odds of exposure to human rights violations by their families. Interestingly, a strong association was found between participants’ reported economic status relative to others and violations by all four perpetrators. Participants who reported fair to poor economic status were more likely to report human rights violations across all scales compared to those who reported very good to excellent economic status. Indeed, the link between human rights violations and poverty is well established in the literature, with poverty described as the ‘worst attack on human rights’ (53–55).

The association with education may be mediated by a greater awareness of rights related to improved education. This remains speculative, although the association persists even after controlling for income as a variable. This requires further research to elucidate such findings. Overall, one must exercise caution in interpreting these results, as it is not possible to ascertain the direction of causality due to the cross-sectional nature of this study. Finally, and sadly, analysis reveals that persons reporting at least one disability are more likely to report human rights violations by their families. While such findings have also been reported in the literature (56), they underscore the need for focused studies on human rights violations among people with disabilities in the future.

## Conclusion

To summarize, this research highlights the importance of including the family and community as potential perpetrators in studies of human rights violations rather than focusing solely on violations committed by governments or occupying forces during international conflicts. This broader perspective offers a more comprehensive understanding of the issue. While violations at the family and community levels may be addressed through policy reforms that strengthen protection laws and provide support services for victims, those committed by the Israeli occupation and the Palestinian Authority are more complex and may require important political changes.

This research also emphasizes the significance of using mixed methods—both qualitative and quantitative—which can bring research closer to the reality in which people live (57). Moreover, qualitative research allows people’s views to influence outcomes by giving them the opportunity to speak for themselves rather than having others speak on their behalf.

While this study does not present our qualitative findings, the questionnaire used and the scales developed are based on that research. Conducting quantitative research after qualitative investigations is beneficial for identifying patterns and priority groups for action and ensuring generalizability. This approach can help reveal how people conceptualize and define human rights violations, contrasting these perspectives with institutional and governmental definitions; it allows for the emergence of diverse ways of knowing (58) and contributes to the decolonization of knowledge production, an essential aspect of the research we and others have conducted over the years.

Whereas an assessment of how human rights violations affect people’s health will be addressed in future research, it is important to emphasize in this study that such violations inevitably impact people’s lives and health, whether in terms of distress (59), the quality of their lives (60), their self-rated health, or their overall wellbeing (61), among other measures of health status. In fact, our lived experiences of persistent violation due to enduring chronic warlike conditions as persons, families, and professionals have led us to conceptualize such violations as part of the realm of suffering (62). Undeniably, the suffering of Palestinians under Israeli military rule and colonization, and likely elsewhere, given the significant presence of various forms of structural violence, is an integral part of daily life and is deeply felt. This suffering produces invisible wounds within a person that, depending on the degree, severity, and chronicity of the violations, as well as the resources available for recovery, may either foster healing and strength—the capacity to endure and resist, which is a more appropriate term for resilience (63) or lead to states of trauma and illness resulting from the violations.

Particularly during wartime, as violence permeates daily life, an (64) ongoing experience for Palestinians, this combination of familial, communal, governmental, and Israeli military violations can be synergistic, intensifying the suffering people endure. This makes life increasingly difficult to live and likely results in severe negative consequences for health, both physical and mental, because, ultimately, health is a social and political construct.

Finally, we hope this study provides important insights into measuring human rights violations experienced by Palestinians while contributing to the broader discourse and research on global human rights violations, particularly in war-like conditions and conflict-affected zones.

## Data Availability

The raw data supporting the conclusions of this article will be made available by the authors upon request.

## Supplementary material

The Supplementary material for this article can be found online at the following link: https://www.frontiersin.org/articles/10.3389/fpubh.2025.1557817/full#supplementary-material
