# Correction: Measuring human rights violations from an ecological perspective using a locally generated instrument: a cross-sectional study of Palestinians in the Israeli-occupied West Bank

**DOI:** 10.3389/fpubh.2026.1803158

**Published:** 2026-02-16

**Authors:** Frontiers Production Office

**Affiliations:** Frontiers Media SA, Lausanne, Switzerland

**Keywords:** human rights violations, community and society, government, Israeli military occupier, measurement, West Bank, Israeli occupied Palestinian territory

Reference 19 was erroneously written given the accession date September 19, 2025. It should be September 19, 2024.

The original version of this article has been updated.

