# Supplementary material for: Measuring human rights violations from an ecological perspective using a locally generated instrument: a cross-sectional study of Palestinians in the Israeli-occupied West Bank
Source: Front Public Health. 2025 May 22;13:1557817. doi: 10.3389/fpubh.2025.1557817 (PMC12137323; doi:10.3389/fpubh.2025.1557817)
Supplement: Supplementary file 1 [file Table_1.docx]

**Annex 1: Human rights violation scale developed by the Institute of Community and Public Health**

1. **Human rights general scale**

**Have you ever experienced deprivation or violation to any of the following rights?**

*Answers : [1] Not at all [2] a little [3]a lot [4] an extreme amount*

1. The right to be treated with respect
2. The right to have your decisions be respected regardless of gender (men or women), age and ideas
3. The right to have your personal freedoms respected
4. The right to safety
5. The right to education
6. The right to freedom of expression
7. The right to health
8. The right to have adequate infrastructure (Sewage network, water network, electricity network, roads network…)
9. The right to be treated with equality and without discrimination
10. The right to movement and mobility without restrictions
11. The right to live
12. The right to work
13. The right to live with freedom
14. The right to maintain your dignity
15. The right to practice political rights without any restrictions

**Scale building :**

- Answers : [1] Not at all [2] a little [3]a lot [4] an extreme amount
- Codes 3 and 4 are recoded to yes with a code of 1
- Codes 1 and 2 are recoded as no with a code of 0
- The scores were summed to get a scale between 0 to 15
- The scale can be used as continuous with a range from 0 to 15 or can be recoded into 0 =0 no violation and 1-15 =1 at least one violation

1. **Human rights violation specific scales**

Any participant who answers 2, 3 or 4 in the general scale, will be asked:

Who deprived you from your right to (*mention the right that was violated*).

**Answers:** [1] Family [2] Society [3] Palestinian Authority (PA) [4] Israeli military occupation (select all what applies)

4 different specific scales can be generated:

1. Human rights violations by the family
2. Human rights violations by the community
3. Human rights violations by the PA
4. Human rights violations by the Israeli military occupation

All of which have scores from 0-15 as continuous or 0-1 as categorical with the following values

1. No violation
2. At least one violation
